# Editorial: Odontogenic Tumors

**DOI:** 10.3389/froh.2022.845557

**Published:** 2022-02-25

**Authors:** Toshinari Mikami, Wilfredo Alejandro González-Arriagada, Sven E. Niklander, Carolina Cavalieri Gomes, Ricardo Santiago Gomez, Ronell Bologna-Molina

**Affiliations:** ^1^Pax Creation Medical Lab, Morioka, Japan; ^2^Department of Oral Pathology, Oral Lab Central College of Stomatology, China Medical University, Shenyang, China; ^3^Facultad de Odontología, Universidad de los Andes, Santiago, Chile; ^4^Facultad de Odontología, Unidad de Patología y Medicina Oral, Universidad Andrés Bello, Viña del Mar, Chile; ^5^Department of Pathology, Biological Sciences Institute, Universidade Federal de Minas Gerais, Belo Horizonte, Brazil; ^6^Faculty of Dentistry, Department of Oral Surgery and Pathology, Universidade Federal de Minas Gerais, Belo Horizonte, Brazil; ^7^Facultad de Odontología, Laboratorio de Patología Molecular Estomatológica, Universidad de la República, Montevideo, Uruguay

**Keywords:** odontogenic tumor, ameloblastoma, malignant odontogenic tumor, genetic mutation, diagnosis, conservative treatment, metabolic pathway

There is always controversy about the boundaries of odontogenic tumor classification, whether a given lesion is a hamartoma, a benign tumor, or a malignant tumor. A true tumor should have mutations or epigenetic abnormalities in genes related to cell growth. Experimental elucidation of the pathogenesis and progression of tumors is essential for correct tumor classification, histopathological diagnosis, and appropriate treatment. However, the establishment of cell lines for benign odontogenic tumors is technically challenging and malignant odontogenic tumors are rare. In addition, since many odontogenic tumors occur within the jawbone, their genetic materials are often damaged by demineralization during the tissue processing for pathological examination. Therefore, it is still important to consider the pathogenesis of odontogenic tumors based on detailed observation of case series, histopathological analysis, protein expression analysis, and the developmental process of normal teeth. The aim of this Research Topic on “*Odontogenic Tumors*” in *Frontiers in Oral Health* was to introduce new insights into genetic alterations and protein expression abnormalities that may be associated with the development of benign and malignant odontogenic tumors, including hamartomatous lesions. This article collection includes a series of seven articles that discuss the pathogenesis of odontogenic tumors from various perspectives.

As odontogenic tumors are derived from cells of the tooth forming apparatus and their remnants [1], when discussing the pathogenesis of odontogenic tumors, it is important to consider the age-related changes in these cells. Therefore, the understanding and contextualization of the age-related changes may be an approach to understanding the biology of the odontogenic tumors. In this context, Bastos et al. assessed age-related changes in metabolic pathways of dental follicles associated with unerupted/impacted mandibular third molars. Formalin-fixed paraffin-embedded dental follicles from young (<16 y.o., *n* = 13) and adult (>26 y.o., *n* = 7) individuals were analyzed by high performance liquid chromatography-mass spectrometry-based untargeted metabolomics. Multivariate and univariate analyses were conducted, and the prediction of altered pathways was performed by mummichog and Gene Set Enrichment Analysis approaches. Dental follicles from young and older individuals showed differences in pathways related to C21-steroid hormone biosynthesis, bile acid biosynthesis, galactose metabolism, androgen and estrogen biosynthesis, starch and sucrose metabolism and lipoate metabolism. These findings highlight the importance of the differences in the activity of metabolic pathways related to advanced age, which is fundamental knowledge that should be taken into account when examining the pathogenesis of odontogenic tumors.

Odontogenic tumors can be classified into three categories according to the tissue of origin: epithelial, mesenchymal, and mixed tumors. However, depending on the stage of differentiation in tooth development, tumors may show various histological characteristics, making classification and definitive diagnosis difficult. Non-calcifying Langerhans cell rich variant of calcifying epithelial odontogenic tumor (NCLC-CEOT) and amyloid rich variant of central odontogenic fibroma (AR-COF) share many similar clinicopathological features. Tseng et al. clinicopathologically reviewed reported cases of NCLC-CEOT and AR-COF published between 1958 and 2021. The results indicated that they are most likely to be the same disease entity showing overlapping clinicopathological features with COF rather than CEOT.

The identification of genetic alterations, especially those specific to a tumor, is useful not only for clarifying the pathogenesis of a specific lesion but also for the understanding of its biologic features, and for the establishment of a definitive diagnosis. Guimarães et al. performed a detailed revision of MAPK/ERK genetic mutations in benign and malignant odontogenic tumors, and Marín et al. reviewed somatic mutations of ameloblastoma and adenomatoid odontogenic tumor (AOT). KRAS mutations were identified in 75.9% of AOTs, mainly G12V; whereas BRAF mutations were identified in ameloblastomas, mainly V600E. Amaral-Silva et al. immunohistochemically investigated the expression of DNA methyltransferases (DNMTs) and the histone modification H3K9ac, which are epigenetic markers, in dental follicles, ameloblastomas and ameloblastic carcinomas. Interestingly DNMT3B expression was higher in ameloblastomas compared to dental follicles, while ameloblastic carcinomas overexpressed all proteins. Based on these findings, Amaral-Silva et al. suggested DNA methylation and histone modification to play a role in the development, aggressiveness and recurrence of ameloblastomas.

Malignant odontogenic tumors are extremely rare neoplasms. They arise either *de novo* from the tooth forming tissues, their developmental residues or from existing odontogenic epithelial or mesenchymal neoplasms. Marin et al. systematically reviewed 312 articles which included 507 patients with malignant odontogenic tumors and described the clinical and pathological features of the lesions emphasizing problematic areas in diagnosis and classification. The most commonly reported tumor was ameloblastic carcinoma (25.7% of all diagnoses), but there was considerable variation in how and when various diagnostic terms were used. Several misdiagnoses were also reported. Cervical lymph nodes were the most common site of metastasis and the lungs were the most common organ affected with metastasizing ameloblastoma. Overall, 26.8% of patients developed recurrence.

Because odontogenic tumors arise within the jawbone, the treatment, which is surgical in almost all cases, is highly invasive, even in the case of benign tumors or hamartomas. Sometimes extensive and mutilating surgical treatments can drastically affect the quality of life of the patient. Ameloblastoma is one of the most common odontogenic tumors, however, due to its high level of recurrence, the optimal therapeutic management is still controversial. Rocha et al. evaluated the reliability and effectiveness of two conservative surgical therapeutic protocols for the management of ameloblastomas, curettage only and curettage plus cryotherapy. Conservative curettage, even without adjuvant cryotherapy, was found as a valid alternative in the treatment of ameloblastomas. There were few recurrences and acceptable post-operatory complications that could be easily managed than those experienced after wide resections. In the study of odontogenic tumors, the ultimate goal is to establish an optimized treatment method that will benefit the patient.

In conclusion, this Research Topic provides a variety of new insights into benign and malignant odontogenic tumors, ranging from genetic abnormalities to tumor characteristics, diagnosis, and treatment. The papers that comprise this Research Topic have greatly contributed to our further understanding of the pathogenesis and progression mechanisms of odontogenic tumors.

## Author Contributions

TM and RB-M drafted and further revised the manuscript. WG-A and SN critically reviewed the manuscript and significantly improved it. CG and RG have critically reviewed it. All authors listed have read this editorial and approved it for publication.

## Conflict of Interest

The authors declare that the research was conducted in the absence of any commercial or financial relationships that could be construed as a potential conflict of interest.

## Publisher's Note

All claims expressed in this article are solely those of the authors and do not necessarily represent those of their affiliated organizations, or those of the publisher, the editors and the reviewers. Any product that may be evaluated in this article, or claim that may be made by its manufacturer, is not guaranteed or endorsed by the publisher.
